# Reaching hard-to-reach men through home-based couple HIV testing among pregnant women and their male partners in western Kenya: a qualitative study

**DOI:** 10.1186/s12889-020-08878-0

**Published:** 2020-05-19

**Authors:** Daisy Krakowiak, Pamela Makabong’o, Marielle Goyette, John Kinuthia, Alfred Onyango Osoti, Victor Asila, Molly Ann Gone, Jennifer Mark, Carey Farquhar

**Affiliations:** 1 Department of Epidemiology, University of Washington, Seattle, Washington USA; 2 Department of Research and Programs, Kenyatta National Hospital, Nairobi, Kenya; 3 Department of Reproductive Health, Kenyatta National Hospital, Nairobi, Kenya; 4 Department of Obstetrics and Gynaecology, University of Nairobi, Nairobi, Kenya; 5 Department of Medicine, University of Washington, Seattle, Washington USA; 6 Department of Global Health, University of Washington, Seattle, Washington USA

**Keywords:** HIV, Pregnancy, Sexual partners, Women, Men, Counselling, Testing, Intervention, Women, Health systems

## Abstract

**Background:**

Globally only 79% of adults living with HIV (human immunodeficiency virus) know their status and men in sub-Saharan Africa are considered a particularly hard-to-reach population for HIV testing. Home-based HIV couple testing during the antenatal period is a safe and effective method that has been used to test male partners of pregnant women. The goal of this qualitative study was to identify elements that made couple testing successful and describe important characteristics of this home-based intervention from couples’ perspectives.

**Methods:**

Couples who received scheduled home-based couple testing during pregnancy in Kisumu, Kenya, were purposively sampled based on HIV status from January to May 2015. An interviewer administered all of the in-depth interviews and two coders were directly involved in the data analysis and reconciled codes several times in the process.

**Results:**

Twenty-one couples were enrolled: 9 concordant HIV-negative couples, 8 HIV discordant couples, 3 HIV concordant HIV-positive couples, and 1 whose concordance status was unknown. Median age at the time of home-based couple testing was 24 and 28 years for women and men, respectively. Median relationship duration was 3 years and couples had a median of two pregnancies. The major themes that emerged were that home-based couple testing 1) removed the female burden of requesting couple testing, 2) overcame logistical barriers associated with clinic-based testing, 3) encouraged participants to overcome their fear of testing and disclosure, 4) provided privacy in the home, and 5) provided quality time with the health advisors. Importantly, some women appreciated individual testing at the clinic before couple testing and some couples preferred skilled, anonymous health advisors delivering the intervention rather than known community health workers.

**Conclusions:**

The results of this qualitative study suggest that home-based couple testing during pregnancy overcame many of the barriers that limit men’s access to and uptake of clinic-based testing. It encouraged participants to overcome their fear of testing and disclosure through a setting that afforded privacy and quality time with skilled health advisors. These qualitative results may help design effective partner and couple HIV testing programs in the antenatal setting and alongside or within other assisted partner notification services.

**Trial Registration:**

Clinicaltrials.gov registry: NCT01784783. Registered prospectively on June 15, 2012.

## Background

Implementing male partner HIV (human immunodeficiency virus) testing among pregnant couples remains a challenge in sub-Saharan Africa as women often present alone at antenatal appointments [1–3]. However, it is critically important for programs to engage men if they are going to achieve 95-95-95 goals. Furthermore, pregnant women are at an increased risk of acquiring HIV and transmitting the infection to their infants, making male partner HIV testing and prevention during the antenatal period of utmost importance [4–8].

Several qualitative studies have reported barriers to male antenatal clinic attendance, including the perception of antenatal care as a female responsibility, the experience of not being welcome at the clinic by clinic staff and long waiting times, the belief that it is culturally inappropriate as a male to be involved in female-oriented clinic visits, the lack of knowledge of the importance of male involvement, and the fear of learning one’s HIV status [9–17]. Not only is it desirable to test male partners to address their potential impacts on mother-to-child transmission, but higher rates of uptake of prevention of mother-to-child transmission (PMTCT) interventions by the mother are also observed when men attend antenatal services with their partners [18–22].

Quantitative studies support these findings. Two studies in Kenya were only able to HIV test one-third of male partners using written invitations from their female partners to attend the antenatal clinic [23, 24]. Invitations may have improved upon standard of care where only 5.6% of male partners were tested for HIV in the last 12 months in 2015 [25], but more than half of men did not become involved in antenatal visits using invitations only, suggesting that a complementary or alternative approach is needed. Home-based testing has been explored as an alternative or complementary approach to clinic-based testing and invitation-based models of engaging male partners [24, 26–31] and could be utilized alongside or within assisted partner services [32]. The goal of this qualitative study was to explore aspects that made couple testing successful and identify important qualities of this home-based intervention.

## Methods

### Intervention

The quantitative methods and results from the parent study are described in detail elsewhere [23]. Women attending their first antenatal visit at Kisumu County Hospital (September 2013 - June 2014) were recruited for a randomized clinical trial of home-based couple HIV testing versus partner clinic invitation in Kisumu, Kenya. Eligibility criteria included: ≥14 years of age, ≥8 weeks gestation, being married or cohabiting, not having a male partner present at the clinic visit, having partner ≥18 years of age, planning to live ≤40 km from the clinic now until 9 months postpartum, and not having experienced physical, verbal or sexual abuse in the past month.

Of 601 eligible and consented women, 306 women were randomized to receive a scheduled home-based partner education and testing (HOPE) visit within two weeks of enrolment. The other 295 women were randomized to receive a written invitation encouraging the male partner to attend the clinic (INVITE or clinic-based testing by invitation) and delayed home-based partner education and testing at 6 months postpartum. Women in both arms were HIV tested at the clinic following study enrolment and women and their partners were retained in the study until 6 months postpartum for follow-up of outcomes.

Three teams of two health advisors each, one female and one male, were hired for the study. All health advisors had previous HIV testing and counselling experience. For women in the intervention arm receiving home-based testing, an intervention appointment with her partner was scheduled. A home locator visit was done for all couples immediately after enrolment to record the home location for follow-up purposes. Scheduling for the intervention was done in-person if the partner was present at the home locator visit or over the phone if the partner was not present. Phone scheduling was done with the woman or, if the woman allowed and provided her partner’s phone number, with phone contact with the partner. The intervention could take place during the home locator visit if it was convenient for the couple. Intervention visits had flexible scheduling and could take place during early mornings, throughout the day, evenings, weekdays and weekends.

The scheduled intervention visit included introductions, partner consent for participation in the study, pre-test couple HIV counselling, rapid testing, and post-test couple HIV counselling, with individual testing offered to those who were not willing to test as a couple. The visit also included health education on facility delivery, exclusive breastfeeding, postpartum family planning, and preventing STIs (sexually transmitted infections). The intervention took approximately 1-1.5 hours, with slightly longer time needed for discordant couples. Couples were given 300 Kenyan shillings total (about 3 U.S. dollars) for their time.

Male partners were more than twice as likely to have been tested in the HOPE (home-based partner education and testing) arm as the INVITE (clinic-based invitation testing for the partner) arm (87% vs. 39%) by 6 months postpartum per their own self-report. Furthermore, women in the HOPE arm were also twice as likely to know their partner’s HIV status as the INVITE arm (88% vs. 39%); mainly because couples in the HOPE arm were more than three times as likely to have been tested as a couple as the INVITE arm (77% vs. 24%). Additionally, home-based couple testing was more effective at reaching and testing discordant couples, as more were identified and known to women in the HOPE arm than the INVITE arm (13% vs. 4%). All women in discordant partnerships where the man was positive did not know they were in discordant partnerships at enrolment.

### Qualitative study

We utilized a grounded theory approach and purposively selected couples based on HIV status who were nearing study exit at 6 months postpartum and oversampled couples in which one or both were HIV-positive. All participants who were discordant or concordant HIV-positive and nearing their 6 months postpartum exit visit were invited to participate, whereas a random sample of HIV-negative couples were selected in the sample. Our target sample size was twenty couples as we suspected to reach data saturation with this number. As manuscripts were reviewed as new interviews were taking place, we checked whether new themes were emerging as the sample size grew. The plan included sampling more individuals if new themes emerged in later interviews.

A semi-structured interview guide was developed to explore female and male perceptions and experiences of home-based HIV testing, intervention influence on behaviours and outcomes, as well as to collect feedback and suggestions for improvement for the home-based intervention.

Participants were contacted by an experienced local female qualitative interviewer (from Maseno University in Kisumu, Kenya) with a phone call and she introduced herself as someone working with the study they were a part of, and invited them to participate in in-depth interviews. Participants who agreed to meet in person at their 6-month postpartum follow-up with the interviewer had a face-to-face explanation and consent process was administered. Written informed consent was obtained from all individual participants included in the study prior to being interviewed. Participants were provided with the explanation that “the purpose of the interview step study is to find out what you think of the home-based education and testing you received (during pregnancy)” and that the benefit of the study “will help us learn the best way to deliver home-based education and testing to pregnant women and their partners.”

The interviewer administered all of the interviews using a semi-structured, translated guide that was pilot tested with several couples. Pilot testing impacted question order and some phrasing but not the question content. The interviewer administered individual interviews for sensitive topics, such as testing alone prior to testing together for women, and also interviewed couples together for other topics. The interviews were administered mostly in participant’s homes during their 6-month postpartum exit interviews from January to May 2015 and took approximately 1-1.5 hours to complete. The interviewer was the only one present during the interviews with the couples, both individually and together, along with any small children the couple had, and no repeat interviews were administered. The interviews were recorded using digital recorders, transcribed, and translated into English from Luo and Kiswahili by the same person who conducted the interviews with field notes being made during the interview on the interview guide. Transcripts were not returned to participants for comment and/or correction.

English transcripts were imported into ATLAS.ti version 7.5.10 (Berlin, Germany) for analysis. Two coders, the qualitative principal investigator and an experienced qualitative coder, were directly involved in the data analysis. Codes were derived from the data and the coders reconciled codes between them several times in the process to create a coding tree. Quotes were identified as being representative of those themes and patterns in the coding tree and are presented in the text and tables. Participants did not provide feedback on the findings.

This research was approved by the University of Washington Institutional Review Board and Kenyatta National Hospital Ethics Review Committee.

## Results

### Participant characteristics

Of 30 couples who were reached by phone, 21 couples completed in-depth interviews: 9 concordant HIV-negative couples, 8 discordant couples (5 female HIV-positive, and 3 male HIV-positive), 3 concordant HIV-positive couples, and 1 whose concordance status was unknown (female HIV-positive, male not tested). The 9 couples who were reached but were not interviewed were due to the following reasons: 3 had partners who were away (not in town for work or other reasons) so could not participate as a couple, 2 couples had separated, one individual said they did not have time, one individual did not return calls for a rescheduled interview, and one couple had moved out of town. While comparisons between interviewed couples of different serostatuses were not appropriate due to still a relatively small sample size in each serostatus category, our goal was to capture narratives from each type of couple to inform the overall results of the study. While themes cannot be derived for each serostatus category, serostatus of couples is included in quotes and some explanations to provide context. Data saturation was reached with the number of couples interviewed and no new themes emerged in later interviews.

The median age at the time of intervention for women was 24 years and 28 years for men. Median relationship duration was 3 years and couples had a median of two pregnancies. There were no polygamous relationships in the sample. The median education level for women was upper primary grades 4-8 and for men was secondary school completion.

### Overview of themes

In the analysis, five major themes emerged (presented in chronological order of the testing experience, not necessarily in order of importance). Home-based couple HIV testing: 1) removed the female burden of requesting couple testing, 2) overcame logistical barriers associated with clinic-based testing, 3) encouraged participants to overcome their fear of testing and disclosure, 4) provided privacy in the home, and 5) provided quality time with the health advisors. Lastly, women commented on the usefulness of being tested alone beforehand and their preference of skilled, anonymous health advisors.

#### Removed the female burden of requesting couple HIV testing

A major theme discussed by women is that home-based couple testing overcame the female burden of trying to get their partner tested at the clinic. With the health advisors contacting, scheduling and visiting the couples in their own homes, women no longer had to do this on their own. One woman expressed the home testing “*was good because I have pleaded with him to go for the test and he refuses, so when you came here I was very happy because he had to”* (concordant negative female, participant couple 6).

Another woman explained that home testing “*is good because it is being done with your partner because sometimes your partner doesn’t want to go to the hospital, so when they come home the test is done together and the result is out”* (concordant negative female, participant couple 3).

#### Overcame logistical barriers associated with clinic-based testing

Some men cited they were too busy to go to the clinic, including not being able to take time off work to attend the clinic when it was open, and were more willing to participate in a scheduled home visit due to convenience, saved cost on transport and being held more accountable with a scheduled appointment. One man explained that he *“might not have time to go to the hospital, so when you make an appointment to come it is okay”* (discordant negative male, participant couple 14).

#### Encouraged participants to overcome their fear of testing and disclosure

Fear, particularly fear of HIV testing, was the most commonly cited reason as to why men were reluctant to go to the clinic (see Table 1 for additional quotes). One man explained:

*It [home testing] is a good method. That is the only way you could reach out to those who are afraid […] she brings you along the way my wife did and you found me in the house because most men do not escort their wives to the clinic that is the only way you could reach the other villagers when their wives are expecting* (concordant negative male, participant couple 6).

**Table 1:** Fear and logistic barriers of attending clinic supporting quotes

“It [home testing] is not bad because sometime somebody is afraid of going to the hospital so you can find them at home and help them early” (concordant positive female, participant couple 17).	
“There are some people who are afraid of going to the hospital, it is easier when you come to my doorstep unlike the hospital, because they do not incur any expenses like transport” (discordant negative female, participant couple 18).	
“Okay, it [home testing] is still fair according to me, it is also good for those who find it difficult to go to the hospital, so when they come to know about it, they can have a change of mind [then test]” (discordant negative male, participant couple 1)	

Many men and some women were afraid of personally being found HIV-positive. With home-based couple testing, this fear eased once health advisors explained what HIV is, how it can be managed successfully, and the benefits of knowing one’s status. The training and education of the health advisors in this intervention, as well as the time spent with each couple, were of a more focused and specialized level than antenatal nurses performing couple testing at the clinic, where couple testing is an infrequent occurrence as most men do not attend with their partners. In addition to the following in-text quotes, Table 2 provides additional supporting quotes.

**Table 2:** Encouraged participants to overcome fear of HIV testing & disclosure supporting quotes

“It was difficult at first but after the doctor told us about the advantages of the test for a while I agreed, but I don’t usually like” (concordant negative male, participant couple 9).	
“It was good [being involved] because there are some things you cannot just talk about but it will force you to talk about them like when I was asked about my status which I do not like to discuss it with people but I discussed it freely with researchers who came” (discordant positive male, participant couple 18).	
“I was afraid for the first time but after the teaching I was courageous. [And after testing] I felt good because the results were negative and we were both there” (concordant negative female, participant couple 6).	
“I found that first of all you bring awareness because you make people feel free, so what I first put in mind as a human being is that I will not live forever, and I know that diseases kill, but now if I can find something to sustain me in life that is good isn’t it?” (concordant positive male, participant couple 2).	

One man expressed how people can overcome this fear with quality counselling:

*People are afraid of test, though if you approach somebody and request to do for them the test at home and counsel them properly they might agree […] If somebody comes and tell you the benefits of it you will realize it is good to know whether you have it or not to be able to live a longer life* (concordant negative male, participant couple 8).

Another man very much appreciated the skill and friendliness of the health advisors as he explained, “*you talked to us well, your approach was better even the fear disappeared after you introduced yourselves”* (concordant negative male, participant couple 6).

Respondents also appreciated that couple testing was a less daunting disclosure method than disclosing without the help of a health advisor. One man stated that he thought this was good because *“there are some people who don’t disclose their status to their spouses so if you are tested together the woman knows and the husband also”* (discordant positive male, participant couple 18).

Many men and women also expressed fear of being HIV-positive and their partner HIV-negative. As part of pre-test counselling, health advisors asked couples to discuss the different scenarios of what they would do if they were both found to be positive, negative or if only one of them was positive and worked out solutions to different scenarios. When asked what he would have felt if one or both of the results were positive, one man expressed that *“they taught us first before, that such thing happen and what we will do even if we have it so I went in confidently, with knowledge”* (concordant negative male, participant couple 6).

For some couples, post-test counselling was particularly important in relieving the fear and worry of their discordant serostatus. Being asked about how it felt being part of the visit, one man responded that *“it was easy, I have had it easy considering the thoughts I had before this visit, after we were tested and knew our HIV status, we had many thoughts, but after the education we got from the visit, all these days, I lost the thoughts and felt light”* (discordant negative male, participant couple 1).

Furthermore, most concordant positive, negative and discordant couples spoke at length at how their relationships improved following disclosure, including increased trust, improved support, and better communication. For concordant negative couples, their shared negative HIV status served as a proxy of having no outside partnerships and reaffirmed the commitment they had to one another as they agreed to not have external partners. For discordant couples, their acceptance of their discordant status seemed to also improve upon their relationship as they resolved to stay together and help one another in preventing and managing HIV. For concordant positive couples, a similar phenomenon of acceptance and peace as well as moving forward in order to live well was noted (see Table 3 for supporting quotes).

**Table 3:** Improved relationship following disclosure supporting quotes

“They [the teachings] helped us because I saw so many differences. Because if you have your husband and you don’t know your status, he would sometimes walk out and move around and after knowing our results we decided to maintain one partner each” (concordant negative female, participant couple 4).	
“I did not see anything bad [in the visit], it was all good. First of all, I thought it would make us break up with my wife, but my wife also encouraged me, and encouraged the relationship because we have children so we should look at the life ahead, so there is nothing bad” (discordant positive male, participant couple 21).	
“I found it easy and it also gave us an opportunity to be at peace with each other in our house, because I know her status and she also knows mine, what remained is us being together and remind each other, I was not jealous in any way, when she is supposed to take her medicine I remind her” (discordant negative male, participant couple 1).	
“It [the visit] has brought a good one [difference] because we are now staying with peace because everyone knows each other’s status so we live well” (concordant positive female, participant couple 17).	
“There was some peace because all of us had so they will not accuse somebody else that you are the one who has given him” (concordant positive male, participant couple 5).	

#### Provided privacy in the home

Another reason that couples preferred home-based testing was that they found it to be more private than clinic settings. As expressed by a female participant:

*The experience was nice, because you know you are free, you are in the house, you know at the hospital you fear those who are there, the nurses and the doctors, you will fear they may know that you are positive, or know your status and maybe they know you they will go to advertise, unlike in the house* (concordant negative female, participant couple 11).

A man likewise stated that *“testing at home is good because it finds you are in a private place, only you, your wife and those who test, there are no other people who hear you, I found it good. The one at home was very good because we were free as compared to the hospital”* (discordant negative male, participant couple 21).

#### Provided quality time with health advisors

Quality time with the health advisors was another a major benefit of home compared to clinic-based testing. A man expressed privacy in the home as being a catalyst to being able to have quality time as he described “*in the hospital people are many and somebody is afraid so this one [home testing] is good because everybody takes his time to listen.’* (concordant positive male, participant couple 5)

Health advisors having more time to spend in the home versus hospital setting was also mentioned by many women and men. A woman stated that the health advisors “*have the time to sit down and teach you, unlike the hospital which is just testing and you go, they do not sit with you down”* (discordant positive female, participant couple 15).

### Usefulness of women testing alone at clinic prior to couple testing

Most women tested alone at the clinic prior to being tested together with their partner (not disclosed to partners by health advisors). For some women, having tested beforehand at the antenatal clinic brought some relief, particularly if found to be negative, and if positive, they felt more prepared going into couple testing. However, testing before at the clinic does not seem necessary for some women, particularly those who are found to be HIV-negative, but is an important step in the process for others. Most women did not disclose individual status to their partners prior to couple testing.

### Health advisor preferences

There were mixed preferences of having one or two health advisors conduct the visit, with some saying it is better to have one for clarity and increased privacy and others saying two were better for completeness of teaching and ability to have mixed gender health advisors.

In general, women preferred female health advisors but men had mixed opinions on gender with some men preferring male health care workers due to relatability and some preferring female health workers due to perceived gender applicability and their personal experience on maternal health topics. Most men said if only a female health advisor could be present, it would be fine as well if the teaching was the same high quality. One man stated, “*I cannot lie about that because even if I say that they should be men because I am a man, I think that will be a mistake, so whether it is a woman or a man provided s/he does the visit, and the teaching they have brought is what is important”* (discordant positive male, participant couple).

However, both men and women strongly preferred having a non-local health advisor, rather than someone from the community (a local community health worker or midwife), conduct the visit due to privacy concerns and some concerns of a lower quality of education provided. The health advisors hired for the study had previous HIV testing and counselling experience and had at least some education beyond secondary school. Only a few men stated they would agree to be visited by someone based locally. One man expressed:

*Those from the community will just help you with advice but they cannot give you much information like those from the study tell you. I would not like someone from the community, because they come out with it openly. They would put it in a different way to spoil your name. I prefer someone who comes from a place where I don’t know [a stranger]. Someone who knows you will speak about it [your status] to others but those who come from outside [far from the community] will keep your secret* (discordant positive male, participant couple 21).

### Individual and dyadic interviews

By and large, individual and couple interviews yielded the same themes, complemented one another, and did not provide discrepant information or results. The benefit of speaking to individuals alone is that it provided the opportunity for one person to express himself or herself to a fuller extent without inadvertently being interrupted by a partner and for us to determine if what was said in an individual interview agreed with what was said as a couple. The benefit of speaking to the couple together was that they were given the opportunity to explain how they make decisions as a couple.

## Discussion

The results of this qualitative study suggest that home-based couple testing during pregnancy overcomes many barriers associated with clinic-based testing including the female burden of requesting couple testing and the logistical barriers of attending clinic as well as encourages male and female participants to overcome their fear of testing and disclosure. Contributing factors include that the setting afforded privacy and couples had quality time with skilled health advisors. Overall, couples preferred home testing to clinic testing and appreciated being tested and educated together as a couple rather than as individuals.

Home-based couple testing has been found to be acceptable and favoured by clients to clinic-based testing in other studies [24, 26–31]. In the same province in Kenya, a qualitative study found that couples strongly favoured couple HIV testing with a trained health worker as a way to disclose status, and that home testing was equally acceptable as clinic testing in this rural setting with an introduction of service into the community, the training of health workers, and confidentiality [31]. Likewise, in Lesotho, a qualitative study reported that both women and men found the home to be a supportive and comfortable environment for testing couples together [28]. Furthermore, in a qualitative study in Uganda, fear was identified as a major barrier to clinic-based testing among pregnant couples [33].

Not only has home-based testing been found to be acceptable in qualitative studies, but in quantitative studies as well; in pilot studies in Kenya [24, 30] and Malawi [26], couples accepted home-based couple testing and counselling at high rates (85%, 64% and 87%, respectively), and in the same Kenya study [24], the majority of men (81%) and women (65%) participants recommended home testing compared to alternative HIV testing venues [29]. These high rates are replicated in the larger parent study [23], where nearly ninety percent of men were tested in the home-based testing arm compared to about forty percent of men who received a clinic invitation (87% vs. 39%, respectively).

Due to the variable feedback on gender preferences, programs may want to accommodate preferences of each couple. If that is not possible, female health advisors were generally preferred. Sending one or two health advisors could also be informed by a priority-based model, where women who are HIV-positive, who anticipate issues in testing and disclosure and/or suspect their partner is positive are visited by two health advisors. Additionally, female preference on whether testing alone prior is important to them should be taken into account. Couples were also asked if they thought other couples would participate in the intervention without study compensation (300 Kenyan shillings per couple, approximately 3 U.S. dollars), and most stated that the education was valuable in and of itself and no compensation was needed. Programs should also plan to conduct visits during times when the partner is not at work, such as early mornings, evenings, and weekends. Health advisors also noted the importance of being in plain clothes and not using marked work bags associated with HIV.

Lastly, while home-based testing was overall preferred to clinic-based testing, some couples chose to have their intervention visit with their partner at the program office at the hospital and this location preference could also be accommodated when possible. This flexible preference-based or priority-based model may also include invitations with phone and in-person follow-up to attend the clinic, as both have also demonstrated effectiveness in reaching men in two recent studies [34, 35]. These methods may be included as part of a comprehensive strategy of male partner involvement, starting with invitations, followed by phone tracing and scheduled home-based visits if not successful.

This qualitative study had several strengths including interviewing both women and men, being nested within a randomized clinical trial and asking about an actual intervention the couple had received rather than hypothetical scenarios. By including both men and women ensured, both sides of the experience were captured rather than relying on women or men to serve as proxies for each other. There is potentially less sampling bias in the qualitative study because couples were randomized to the intervention and did not choose the intervention. Also, the participation rate in interviews were also high for this type of qualitative study. The qualitative study captured responses regarding an intervention the couples had, rather than asking about a theoretical intervention. The qualitative in-depth interviews also provided the presented feedback on potential modifications to the intervention, most importantly on the type of health worker, which will be useful in future scale-up implementation studies.

A limitation of our study includes not interviewing those who had not received the intervention, although we have quantitative data on their outcomes and behaviour. A high participation rate of couples in the home-based intervention motivated us to capture responses of this group to learn more about why home-based testing was so successful, so as to insure the integrity of the program in future scale-up. However, a future study determining what else could be done to reach those 10-15% of couples not reached with the home-based intervention would be valuable. Additionally, interviewing men who responded to clinic invitation and attended would be useful to explore how this method works for some men. Another potential limitation to our study is limited generalizability, as both the qualitative and quantitative study was conducted in one peri-urban site in western Kenya and results may differ by setting, including more urban vs. more rural locations.

## Conclusions

Qualitative understanding of interventions aimed at engaging and testing male partners during pregnancy is still lacking. This type of qualitative data and feedback would be useful to include in research and programs planning to implement these interventions, particularly alongside other partner notification services so as to optimize such interventions. Antenatal programs should consider engaging male partners with scheduled home-based testing in order to make couple testing and disclosure possible.

## Data Availability

The qualitative datasets generated and/or analysed during the current study are not publicly available to protect participant confidentiality of full interviews which contain personal and potentially identifying details of individuals.

## Abbreviations

HIV: Human Immunodeficiency Virus
HOPE: Home-based Partner Education and Testing
INVITE: Clinic-based Testing by Invitation
PMTCT: Prevention of Mother-to-Child Transmission
STIs: Sexually Transmitted Infections
